# GP roles in emergency medical services: a systematic mapping review and narrative synthesis

**DOI:** 10.3399/BJGPO.2023.0002

**Published:** 2023-05-31

**Authors:** Alexander Burrell, Grace Scrimgeour, Matthew Booker

**Affiliations:** 1 Centre for Academic Primary Care, University of Bristol, UK; 2 Bristol Medical School, University of Bristol, UK

**Keywords:** community care, systematic reviews, emergency medical services, general practitioners, primary healthcare

## Abstract

**Background:**

A significant proportion of emergency medical services (EMS) work is for problems that may be amenable to timely primary care management and could benefit from GP input. Utilising GPs in EMS may reduce avoidable emergency department (ED) conveyance, releasing emergency ambulances for higher-acuity care, and meeting patient needs earlier in the evolution of an emergency call.

**Aim:**

To collate and summarise evidence on how GPs are utilised in EMS.

**Design & setting:**

Systematic mapping review and narrative synthesis.

**Method:**

A systematic literature search was conducted using search terms for general practice and emergency care. Primary research articles investigating the utilisation of GPs in non-critical EMS were included. An inductive framework was used to structure the results alongside a narrative synthesis.

**Results:**

Twenty-one articles were included. GPs were embedded in EMS for urgent management of high-acuity patients or used as an intervention to avoid unnecessary ED conveyance in selected lower-acuity patients. The importance of interprofessional relationships and training for GPs involved in EMS was highlighted. No studies explored patient-reported outcomes. Outcomes measured were predominantly ED non-conveyance and admission avoidance, with GP services as an intervention reducing the likelihood of these outcomes.

**Conclusion:**

Embedding GPs in EMS might service different purposes depending on context. There is some evidence that GP EMS services may reduce the likelihood of ED conveyance and hospital admission in selected cases; it is unclear whether this is owing to case selection or GP involvement. Future research should incorporate patients’ views and experiences.

## How this fits in

With professional expertise in the management of undifferentiated health problems in the community, utilising the skillset of GPs in EMS may avoid patients being conveyed to the ED unnecessarily. This systematic mapping review, the first of its kind, describes how GPs are integrated into EMS in health systems and have been used by some ambulance services as an intervention with the aim of reducing conveyance to ED and avoiding hospital admissions. It is not clear whether it is the selection of specific low-acuity cases or the involvement of GPs that may reduce the likelihood of ED conveyance. While there appears to be context-specific clinical benefit to GPs in EMS, future research should explore how to optimise models that balance system and patient perspectives.

## Introduction

In England, demand for ambulances and emergency 999 calls are increasing,^1^ as are ED attendances.^2^ The ambulance service no longer exists purely to convey acutely unwell patients to ED. The majority of cases encountered are not immediately life-threatening injury or illness, and problems that may benefit from primary care input represent a substantial proportion of the pre-hospital workload.^3^ There are myriad reasons why primary care problems present to ambulance services, including patients’ social circumstances, complex determinations of urgency, and both perceived and real primary care access challenges.^4^ Ambulance care is a finite resource currently subject to unprecedented demand, and delays in response to emergency calls can cause harm at both individual patient and system level.^1^ However, simply labelling ambulance use for primary care problems as ‘inappropriate’ is problematic; this label is neither sensitive to the patient context nor does it offer practical solutions.^5^

Managing primary care problems in EMS presents several issues: firstly, identifying which patients do not require conveyance to ED. While paramedics are recognised to have skillsets that bring value to primary care,^6^ there is evidence to suggest that paramedics who have not been specifically trained in the assessment and management of urgent care or primary care presentations may experience challenges regarding conveyance decisions in these groups.^7^ This may lead to either unnecessary ED conveyance, or patients not benefitting from access to a senior clinical decision. Second, if patients are appropriately identified as not requiring ED conveyance, their presentation still requires (often urgent) management. It stands to reason that GPs, who regularly manage risk and uncertainty in ‘diagnostic-test light’ settings and share this risk with patients, would be best placed to manage primary care problems. Utilising GPs in EMS may be an effective way to reduce avoidable transfer to ED, release emergency ambulances for higher-acuity care, and provide the most appropriate level of care for patients. Some ambulance services in England have started engaging GPs to provide frontline crews with easy access to primary care expertise and support. However, evidence for GPs in EMS in general or to support a particular deployment approach is lacking. This systematic mapping review and narrative synthesis therefore aimed to collate and summarise evidence on how GPs are utilised in non-critical EMS.

## Method

A systematic mapping review was undertaken of published primary research and grey literature exploring the following question: 'In what ways are GPs utilised in non-critical EMS?' Mapping reviews aim to systematically describe the nature and coverage of literature in a particular area, to inform future in-depth work.^8^ This approach is of particular use in summarising and arranging a wide and heterogenous evidence base.^9^

### Search strategy

The following databases were searched in May 2021 for articles published from January 1990–May 2021: MEDLINE, Embase, PsycINFO, Allied and Complementary Medicine Database (AMED), Cumulated Index to Nursing and Allied Health Literature (CINAHL), Scopus, and Web of Science. Google Scholar was searched pragmatically to identify literature not indexed in the listed databases. Grey literature, book chapters, and theses were searched using OpenGrey, EThOS, and Dart. The resources searched were selected after discussion within the research team and with a subject librarian. Search terms were broad and inclusive, using MeSH and free-text synonyms for general practice and emergency care combined using Boolean operators. The review protocol and search strategy were published prospectively in the PROSPERO register (reference CRD42021242244). An updated search was performed in August 2022 before data extraction. The full search strategy is available in Appendix 1.

### Inclusion and exclusion criteria

The inclusion criteria were primary research articles published in English between January 1990 and August 2022 investigating the utilisation of GPs in non-critical EMS. There were no restrictions on study design, perspective, or outcome measures used. Exclusion criteria were as follows: studies where the main staff group utilised were not GPs; studies investigating purely critical pre-hospital care; studies that reported purely on attendance to the ED or in-hospital care; or studies reporting only the management of one specific clinical intervention, for example, thrombolysis.

### Reference screening

**Figure 1.**
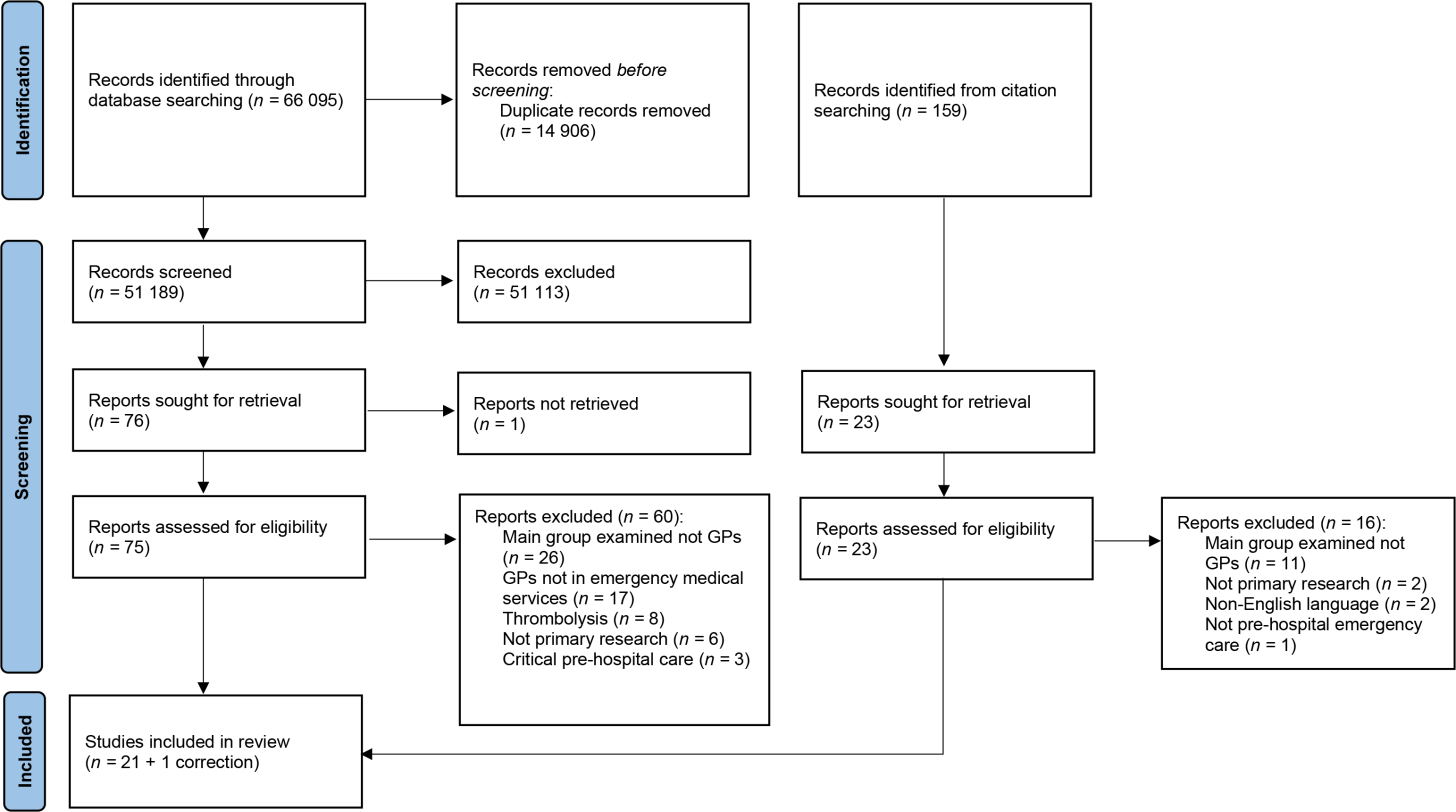
Preferred Reporting Items for Systematic Review and Meta-analysis (PRISMA) flow diagram

References were managed using referencing management software EndNote (version X9.2). The searches, initially performed in May 2021 and updated in August 2022, identified 66 095 references. After duplicate suppression, 51 189 records remained. These were screened by title and abstract by AB from May 2021–August 2022 to exclude obviously irrelevant articles, with MJB reviewing a sample (10%) of these independently with full agreement. The remaining full articles (*n* = 75) were read and reviewed against eligibility criteria by AB and MJB. Forwards and backwards citation searches of accepted papers were performed and screened as above. In total, 21 articles and one correction were included in the systematic mapping process. The PRISMA flow diagram is shown in Figure 1.

### Data extraction

To enable data extraction across heterogenous study designs, a customised data extraction tool was used based on a tool used in previous work by MJB.^4^ Author, date, and citation data were extracted as well as details of the setting, methods, perspective, and main relevant qualitative or quantitative results. Up to three ‘key messages’ were extracted from each study’s discussion and conclusions. AB and GS performed data extraction independently, with disagreements resolved by consensus discussion.

### Quality assessment

For this review, each study was assessed using the relevant version of the Critical Appraisal Skills Programme checklist to enable a consistent approach to internal and external validity across different qualitative and quantitative study designs. Studies were assigned one of the following five categories as used in Dixon-Woods *et al*:^10^ key, satisfactory, unsure, fatally flawed, irrelevant. Those studies that were fatally flawed or irrelevant were excluded from the narrative synthesis. To be fatally flawed, studies had to have considerable methodological limitations such that either the findings could not be trusted, or the conclusions drawn did not match the data provided. Two reviewers assessed quality with consensus discussion to resolve any disparities.

### Framework analysis and narrative synthesis

To describe the literature after data extraction, an inductive framework was developed with an approach used in primary applied qualitative research.^11^ This involves the following five stages of data analysis: familiarisation; identifying thematic framework; indexing; charting; mapping and interpretation. A narrative synthesis, a textual approach to synthesis that summarises and explains findings of included studies, was then conducted based on guidance from Popay *et al* ^12^ to incorporate variability in methodology, outcomes, and contextual heterogeneity.

## Results

Twenty-one articles^13–33^ and one correction^34^ were accepted for data extraction and quality appraisal. The characteristics of these articles are summarised in Table 1. Two studies were felt to be fatally flawed and therefore not included in the narrative synthesis.^13,14^ The full data extraction table is available in Appendix 2.

**Table 1.** Summary characteristics of articles included in data extraction and quality appraisal

Characteristic		Frequency, *n*(% of total)
Method	Qualitative	5 (24)
	Quantitative	13 (62)
	Mixed methods	3 (14)
Study setting	Norway	11 (52)
	UK	4 (19)
	Sweden	3 (14)
	Belgium	1 (5)
	Switzerland	1 (5)
	New Zealand	1 (5)
Year of publication	1990–1999	1 (5)
	2000–2009	4 (19)
	2010–2019	13 (62)
	2020–2022	3 (14)
Quality assessment	Fatally flawed	2 (10)
	Satisfactory	13 (62)
	Key	6 (29)

**Figure 2.**
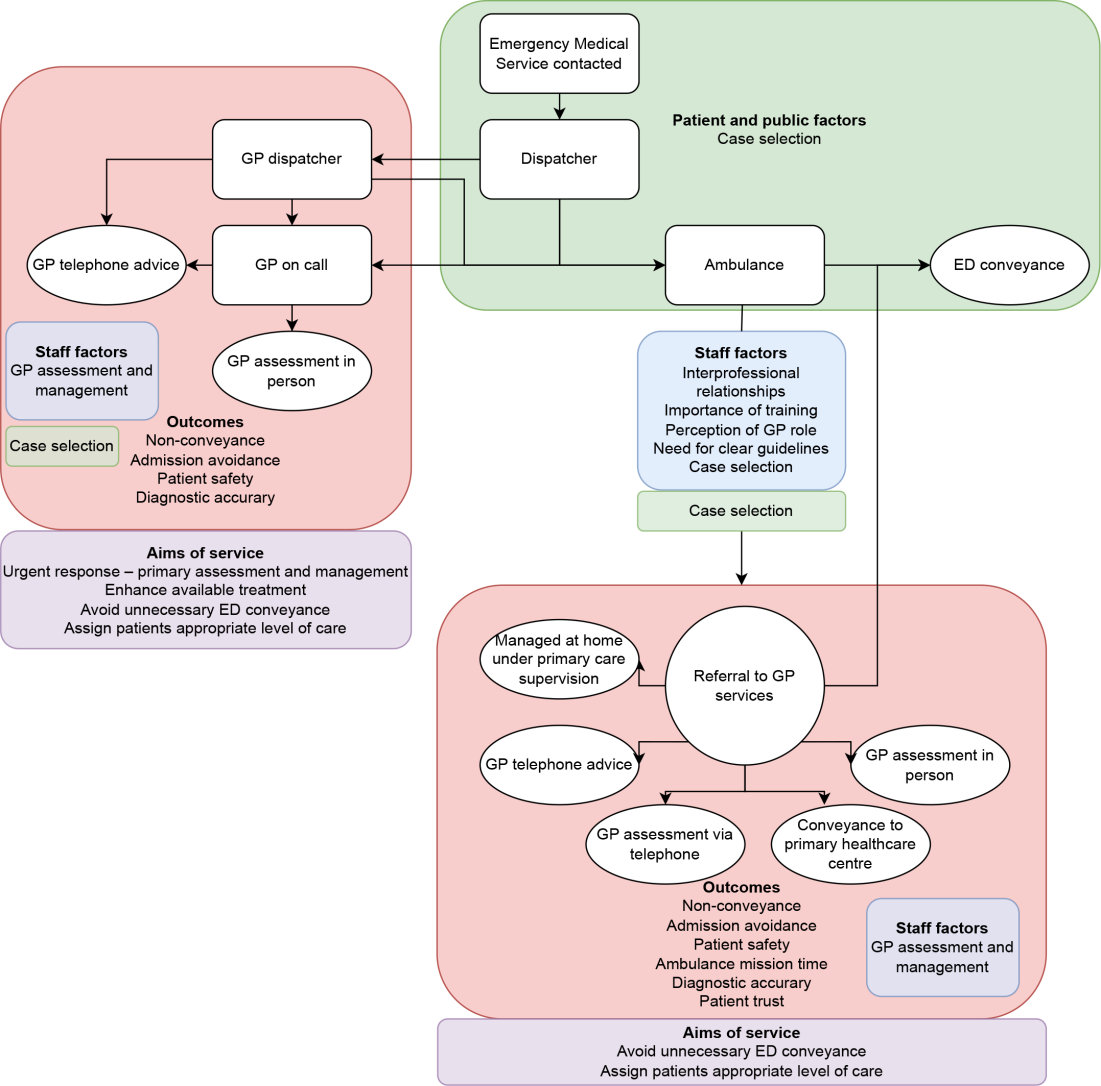
Diagrammatic representation of categories and subcategories in framework. ED = emergency department

Framework analysis led to five categories being identified, with subcategories in each. These are summarised in Table 2 and presented as a process map in Figure 2, as this best and most logically illustrated the included studies and their key messages within the framework.

**Table 2.** Categories and subcategories of evidence developed from the mapping process

Category (number of articles)	Subcategory (number of articles)
Utilisation method (21)	GP dispatcher (1)GP attends directly from dispatch (3)GP alerted by dispatcher — GP discretion whether to attend or provide advice (9)Referral to GP by ambulance staff for management at home under supervision of primary care (1)Referral to GP by ambulance staff for transport to primary healthcare centre (2)Referral to GP by ambulance staff for face-to-face consultation (3)Referral to GP by ambulance staff for telephone support (2)
Aim of service (8)	Avoid unnecessary emergency department conveyance (3)Urgent response — primary assessment and management (1)Assign patients appropriate level of care (3)Enhance available treatment options (1)
Staff factors (31)	Interprofessional relationships (6)Importance of training (6)Perception of GP role (4)GP clinical assessment and management (5)Need for clear guidelines (2)Case selection — staff factors (8)
Patient and public factors (6)	Case selection — patient and public factors (6)
Outcomes (18)	Non-conveyance (5)Admission avoidance (4)Patient safety (4)Ambulance mission time (2)Diagnostic accuracy (2)Patient trust (1)

### Utilisation method

Studies reported a multitude of ways in which GPs are utilised in pre-hospital care. In Norway, GPs are fully integrated into EMS: for patients who are triaged as in a life-threatening situation, the dispatcher alerts both the local GP on call and the ambulance service. Regulation is that both then attend the medical emergency; however, studies suggest GPs play an active role in only 30–50% of these callouts.^16,27^ GPs in Norway also work alongside anaesthetist or emergency physician-staffed helicopter EMS and rapid response cars.^22,31^ The Primary Response In Medical Emergencies (PRIME) scheme in rural New Zealand was the only other system in the accepted articles in which GPs were fully integrated into EMS. If an ambulance crew is more than 20 minutes away, PRIME GPs are called by dispatchers in a medical emergency to assess a patient in person.^20^

Studies investigating GPs in EMS as an intervention were focused on lower-acuity patients. Two studies in England were identified. In the North West Ambulance Service, GPs were integrated via an Acute Visiting Scheme (AVS), whereby paramedics could refer patients triaged as needing urgent or community-supported care to AVS GPs who could then either accept the patient into their care and review in person, or advise the crew to convey the patient to ED.^18,19^ In the West Midlands Ambulance Service (WMAS), on-call GPs employed by WMAS could be directly dispatched to assess and manage patients, or could be referred to by paramedics for face-to-face attendance or telephone advice. Patients had to meet locally agreed triage criteria to be eligible for the GP service, which were *'considered most suited to GP supported assessment'*.^26^ A Belgian study utilised GPs as both EMS dispatchers, who could provide advice to the ambulance team they were dispatching, and as an on-call GP available to both GP and non-GP dispatchers to directly attend cases *'that are considered to belong to general practice'*.^23^ In a Swedish study, ambulance nurses could refer patients who were triaged as green, the lowest acuity, to a primary care physician. They would then decide together on the following appropriate level of care: stay at home under the supervision of primary care; transport to the primary healthcare unit for assessment; or transport via an ambulance to the emergency ward.^21^

### Aims of service

There were four main underpinning a priori aims of involving GPs in EMS:

1. To avoid unnecessary ED conveyance^18,19,26^
2. To enhance available treatment options^26^
3. To assign patients the appropriate level of care^21,23^
4. To provide urgent primary assessment and essential resuscitation to patients.^20^

### Staff factors

Interprofessional relationships with GPs, and perception of their role in EMS were explored with both positive and negative perspectives. Emergency medical technicians in the Norwegian system welcomed the contribution of GPs as a leader and a diagnostic supplement in cases that went beyond guidelines, but found GPs who did not respond to emergency callouts, or were not interested or engaged in emergency medicine, a hinderance.^17^ Paramedics in the AVS also reported frustration in GPs not seeing patients and declining referrals without a rational reason, and felt a lack of respect from GPs.^28^ The need for trust between ambulance staff and GPs in sharing responsibility for patient care was highlighted, and developed over time with respectful encounters.^29^

The importance of training for GPs involved in EMS was prominent. Norwegian GPs who took part in regular multidisciplinary team training were more likely to take part in ambulance callouts. Emergency medical technicians felt such training should be mandatory for GPs in EMS, and GPs appreciated the opportunity to train together.^15–30,30^ Based on cases encountered by GPs in rural EMS, where GPs in Norway are more likely to play a role than in urban areas,^25^ a Norwegian study suggested that GPs working in EMS should be competent in '*fundamental, practical procedures*'. These included venous cannulation, intravenous drug administration, administering oxygen, and recording and monitoring of an electrocardiogram (ECG).^32^ The most common diagnoses EMS GPs faced in this study were transient ischaemic attack or stroke, heart attack, angina, syncope, ‘*epileptic cramps*’, and alcohol ingestion.^33^ In two Norweigan studies on in-person assessment, GPs downgraded the severity of initial triage in almost half of cases they attended.^24,32^

### Patient and public factors

Case selection for GP EMS services in interventional studies was based on patients either meeting pre-defined case descriptions or being in a low-acuity triage category. The WMAS on-call GP scheme identified 19 case descriptions suitable for GP EMS, including patients with an acute confusional state where transferring the patient to hospital was not in the patient’s best interests, or patients undergoing palliative care whose treatment plan was to remain at home.

Certain groups of patients were more likely to be referred to GP EMS services. Older women with call descriptions for less severe or acute problems were more likely to be referred to the AVS, as were patients with a history of a long-term condition. In the WMAS on-call GP scheme, women aged >75 years were less likely than other patient groups to be transferred to ED following GP involvement.^19,26^ Patients and carers buying in to GP services as an alternative to ED conveyance was reported as imperative by patients and ambulance staff:

*'It is important to come to an agreement … it has to be an interaction between the patient and you. Because if you do not have them with you, it will not work.*' (Ambulance personnel B)^29^

### Outcomes

Non-conveyance to ED and hospital admission avoidance were the most reported outcome measures where GPs in EMS were assessed as an intervention. The AVS successfully diverted 60.9–77.6% of patients from ED including up to 30 days after AVS contact.^18,19^ Ambulance duty cycle time for a GP AVS referral was, on average, 15 minutes less than for missions resulting in direct ED conveyance.^18^ In the Swedish study, patients referred to a GP by the EMS triaging nurse were significantly less likely to be transferred to ED (17.4% versus 53.1%, *P*<0.001) and admitted to hospital (11.4% versus 25.6%, *P*<0.001), with a significantly shorter mean ambulance mission time (86.88 versus 94.12 minutes, *P* = 0.04).^21^ For the WMAS GP supported assessment scheme, only 21% of patients were conveyed to ED compared with 61% for the ambulance service as a whole. Patients who received telephone input rather than a face-to-face assessment from a GP were more likely to be conveyed (odds ratio [OR] 2.14, 95% confidence interval [CI] = 1.69 to 2.72).^26^ Norwegian data showed that when GPs on-call were not alerted, patients were twice as likely to be transported directly to hospital (31% versus 16%).^27^ None of the included studies included longer-term clinical outcomes or validated patient-reported experience or outcome measures, and none reported a health economic analysis.

## Discussion

### Summary

GPs have been utilised in EMS in several ways, both experimentally and naturalistically as part of an EMS system that integrates the community response more broadly across the acuity spectrum. When assessed as an intervention, the aim of having GPs in EMS is commonly based on minimising potentially avoidable ED conveyance and hospital admission for patients who are triaged as lower acuity. In these cases, GP services as part of EMS seem to reduce the likelihood of these outcomes, and reduce ambulance mission time. Where GPs are already part of the EMS system — most notably in Norway, where there is a substantial body of evidence examining their role — they are primarily used for urgent assessment and management of higher-acuity patients, where their medical input is seen as a parallel skillset to other pre-hospital clinicians'.

### Strengths and limitations

The searches used in this review were broad and inclusive, which, although leading to a large amount of irrelevant literature that needed to be screened, identified more relevant literature than was expected, with a range of perspectives. However, half of the studies included were from a single country, which may limit how applicable some findings are to other health contexts and settings. Narrative synthesis is useful for developing a coherent story from methodologically diverse evidence, particularly when the review topic has not yet been explored. However, it is an approach that can be perceived as informal and subject to criticism of a lack of transparency.^35^

### Comparison with existing literature

To the authors' knowledge, this is the first broad review exploring how GPs have been utilised in EMS, and with what aims. There is a growing body of work exploring role diversification of GPs including in emergency settings. Recent studies examining GPs working in or alongside ED in England found that clinical benefit was marginal and highly dependent on local factors. For the GPs themselves however, working in ED offered an opportunity to enhance their transferable skills and work flexibly as part of a portfolio career when core general practice was seen to be unmanageable.^36–38^ GPs in EMS does seem to offer some clinical benefit, although the reduction in ED conveyance and hospital admission in interventional studies may be owing to low-acuity case selection rather than the impact of GPs themselves. Once referred, there is minimal evidence that specific patient groups benefit more from GP involvement than others. The data — although limited — suggest that embedding GPs in EMS might serve different purposes depending on context. This review identified only one qualitative study exploring GPs' attitudes to working in EMS, where maintaining and developing skills was highlighted as a benefit.^30^

### Implications for research and practice

Future research should incorporate patients’ views and experiences, and the views of GPs themselves. Unpacking whether reduced ED conveyance in existing and novel GPs in EMS models is owing to case selection or the involvement of GPs would be methodologically challenging but important to study. Beyond the counterfactual of an ‘admission avoided’, it is also important to identify what high value can be added from embedding GPs in the EMS response, and if this is best targeted at certain patient groups. Healthcare providers establishing new services should be clear on the aims of the service, how they utilise GPs, the importance of interprofessional relationships, and what outcomes are of value. The opportunity cost of GPs not working in core general practice should also be considered.
